# On Cross-Sectional Associations of Leukocyte Telomere Length with Cardiac Systolic, Diastolic and Vascular Function: The Asklepios Study

**DOI:** 10.1371/journal.pone.0115071

**Published:** 2014-12-15

**Authors:** Simon L. I. J. Denil, Ernst R. Rietzschel, Marc L. De Buyzere, Caroline M. Van daele, Patrick Segers, Dirk De Bacquer, Wim Van Criekinge, Sofie Bekaert, Thierry C. Gillebert, Tim De Meyer, for the Asklepios Investigators

**Affiliations:** 1 Department of Mathematical Modelling, Statistics and Bioinformatics, Faculty of Bioscience Engineering, Ghent University, Ghent, Belgium; 2 Department of Cardiovascular Diseases, Faculty of Medicine and Health Sciences, Ghent University, Ghent, Belgium; 3 IbiTech-bioMMeda, Ghent University, Ghent, Belgium; 4 Department of Public Health, Faculty of Medicine and Health Sciences, Ghent University, Ghent, Belgium; 5 Bimetra, Clinical Research Center Ghent, Ghent University Hospital, Ghent, Belgium; University of Glasgow, United Kingdom

## Abstract

**Background:**

Systemic telomere length has been associated with measures of diastolic function, vascular stiffness and left ventricular mass mainly in smaller, patient-specific settings and not in a general population. In this study we describe the applicability of these findings in a large, representative population.

**Methods and Results:**

Peripheral blood leukocyte telomere length (PBL TL) was measured using telomere restriction fragment analysis in the young to middle-aged (>2500 volunteers, ∼35 to 55 years old) Asklepios study population, free from overt cardiovascular disease. Subjects underwent extensive echocardiographic, hemodynamic and biochemical phenotyping. After adjusting for relevant confounders (age, sex, systolic blood pressure, heart rate, body mass index and use of antihypertensive drugs) we found no associations between PBL TL and left ventricular mass index (P = 0.943), ejection fraction (P = 0.933), peak systolic septal annular motion (P = 0.238), pulse wave velocity (P = 0.971) or pulse pressure (P = 0.999). In contrast, our data showed positive associations between PBL TL and parameters of LV filling: the transmitral flow early (E) to late (A) velocity ratio (E/A-ratio; P<0.001), the ratio of early (e′) to late (a′) mitral annular velocities (e′/a′-ratio; P = 0.012) and isovolumic relaxation time (P = 0.015). Interestingly, these associations were stronger in women than in men and were driven by associations between PBL TL and the late diastolic components (A and a′).

**Conclusions:**

In a generally healthy, young to middle-aged population, PBL TL is not related to LV mass or systolic function, but might be associated with an altered LV filling pattern, especially in women.

## Introduction

The progression of acquired cardiovascular diseases (CVD) throughout the human life can typically be tracked by a series of gradual changes in physical, chemical and biological parameters. Levels of systolic blood pressure (SBP), cholesterol, C-reactive protein (CRP), smoking status and sex have all been linked with an increased likelihood of adverse cardiovascular events [1]–[3]. Telomere length (TL), although not used in clinical practice, is one such parameter that has repeatedly been linked with cardiovascular health and disease development [4].

Telomeres are the nucleotide-protein complexes that shield the chromosomal ends from erosion caused by the end-replication problem during cell division and distinguishes them from double-stranded breaks to prevent chromosomal fusion [5]. Throughout the replicative lifespan of cells, their TL will decrease until a critical threshold is reached. Critically short telomeres will typically lead to a cell crisis resulting in senescence, apoptosis or immortalization [6]. TL is of particular interest because it potentially provides a cumulative measurement of stresses throughout life representing “biological age” [4].

Although there is still uncertainty about the mechanism(s) by which telomere biology and CVD pathogenesis affect each other, results from both molecular biology and epidemiology have repeatedly shown significant associations [7]–[11]. The same is true for cardiovascular risk factors such as insulin resistance, hypertension [12], smoking status [13], oxidative stress and inflammation [14].

Systemic TL has also been linked to LV structure and function but mostly in smaller, patient-specific settings and not in a general population [15]–[22]. Shorter TL can be found in heart failure (HF) patients [23], [24] and patients suffering from chronic HF have an increased morbidity if their telomeres are shorter [25]. However, reports on the association between TL and indicators of diastolic dysfunction show conflicting results [18], [19]. Similarly, a positive correlation has been described between LVM and PBL TL [20]–[22], but other studies did not detect a significant association between TL and LVM index or LV hypertrophy [18], [26], [27].

The population-based Asklepios Study offers the advantages of a large sample size and the measurement of numerous potential confounders of TL and CVD. We therefore investigated the relations between systemic TL and proven prognostic parameters [28]–[32] of vascular stiffness, cardiac stiffness, systolic function, diastolic function and ventricular mass, to shed light on the baseline state of these correlations.

## Methods

### Study Population

All data presented in this paper were collected during the first round of the Asklepios study on successful (cardiovascular) aging. The study comprises 2524 subjects approximately 35 to 55 years of age, free from overt cardiovascular disease or other significant pathologies at baseline. The full description of the study design, inclusion criteria, detailed methodology and population baseline characteristics have been published previously [33]. The study was conducted in concordance with the principles of the Declaration of Helsinki. All patients gave written informed consent and the study was approved by the Ghent University Ethical Committee. For the analyses reported here, we used the subset of 2509 patients for which reliable TL and all major TL confounder measurements (age, sex, paternal age at birth) were available (cf. De Meyer et al. [34]).

### Biochemical analyses

All subjects were fasting, had refrained from smoking for at least 6 hours and were screened for active infection/inflammation before blood sampling. Conventional serum parameters were measured using commercial reagents according to the manufacturers' recommendations on a Modular P automated system (Roche Diagnostics, Mannheim, Germany), in an ISO 9002 certified reference laboratory [33]. Coefficient of variation of all tests was <3.0%. These parameters included Interleukin-6 (IL-6), C-reactive protein (CRP), oxidized low-density lipoprotein (ox-LDL), serum uric acid concentrations and brain natriuretic peptide precursor [33].

### Telomere Length

For TL-analyses, whole blood was collected in EDTA tubes cooled to 4°C. DNA isolation was performed within 3 days of collection using the Puregene Genomic Purification Kit (Gentra Systems, Minnesota, USA). The DNA was long term stored at −80°C before TL measurement in duplicate. 5 µg was digested with 5U *Rsa*I and 10U *Hinf*I followed by gel electrophoresis, Southern blotting, radioactive hybridization of the telomeric fragments and weight markers, phospho-imaging and quantification (expressed as kbp: kilo base pairs) [14].

### Echocardiographic and vascular examination

Blood pressure was recorded using bilateral triplicate measurements (1 min intervals) on a rested, sitting subject using a validated oscillometric Omron HEM device (Omron Healthcare Co. Ltd., Kyoto, Japan). Blood pressure values of these six readings were averaged and the mean value of systolic blood pressure (SBP) is used throughout this study. The subjects underwent a resting echocardiographic examination and a scan of the left and right carotid and femoral arteries (VIVID 7, GE Vingmed Ultrasound, Horten, Norway). Left ventricular (LV) internal dimensions were measured at end-diastole (LVEDD) with the area-length method. Sphericity was defined as LV width divided by LV length and is expressed as a percentage. Standard 2-D volumetric methods were used to calculate ejection fraction (EF) from end-diastolic and end-systolic LV volumes and to calculate LV mass (LVM). The LVM was scaled allometrically following the recommendations of Chirinos et al. [35] as LVM/(Height)^1.7^ to account for the effects of both obesity and blood pressure on LVM. We also scaled LVM to the body surface area (g/m^2^) [36].

Other cardiac and arterial measurements included the following: systolic (s′), and early (e′) and late (a′) diastolic septal mitral annulus pulsed wave tissue Doppler (TDI) velocities, pulsed wave Doppler early (E) and late (A) diastolic transmitral flow velocities, E-wave propagation velocity (Vpe) and carotid-femoral pulse wave velocity (PWV). PWV was calculated as follows:
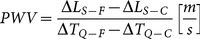
(1)In formula (1) ΔL_S-F_ and ΔL_S-C_ are the distances measured from sternal notch to femoral and carotid measuring sites respectively, ΔT_Q-F_ and ΔT_Q-C_ are the time delays between the start of the QRS complex and the onset of systolic flow in the femoral and carotid artery measured by pulse wave Doppler imaging (full methodology described in the online supplements of Rietzschel et al. [33]). CW Doppler recordings were used to measure isovolumic relaxation time (IVRT) as the interval from the closure spike of the aortic valve to onset of mitral flow.

### Data analysis

Statistical analyses were performed in R 2.15.2. Continuous variables are reported as the mean value ± standard deviation. Means of groups were compared with Student (homoscedasticity) or Welch t-test (heteroscedasticity) as appropriate. To evaluate the contribution of the different confounders to the response variables under study we applied general linear models as implemented in the ‘glm’ function. We report both P-values and the estimated unstandardized effect sizes (b) for TL in these models.

## Results

Baseline characteristics of the population are presented in Table 1.

**Table 1:** Baseline characteristics of the Asklepios study population.

Variable	Women (n = 1291)	Men (n = 1218)	P-value^a^	Population (n = 2509)
Age (years)	45.9±6.0	46.1±5.9	0.316	46.0±6.0
Weight (kg)	66.7±12.7	82.0±12.4	<2.2E-16	74.1±14.7
Height (cm)	163±6	176±7	<2.2E-16^b^	169±9
Body Mass Index (kg/m^2^)	25.1±4.6	26.5±3.7	<2.2E-16^b^	25.8±4.3
Systolic Blood Pressure (mmHg)	123±14	131±13	<2.2E-16^b^	127±14
Pulse Pressure (mmHg)	45.5±9.1	48.3±7.4	<2.2E-16^b^	46.9±8.4
Pulse Wave Velocity (m/s)	6.60±1.45	6.65±1.46	0.397	6.62±1.45
Heart Rate (min^−1^)	67.2±9.5	64.0±10.7	6.01E-15^b^	65.6±10.2
Used Antihypertensive Drugs	145 (11.2%)	118 (9.69%)	0.284^c^	263 (10.5%)
PBL TL (kbp)	7.96±0.73	7.78±0.71	3.26E-09	7.87±0.73
E (cm/s)	78.9±14.2	70.6±13.0	<2.2E-16^b^	74.9±14.2
A (cm/s)	63.5±11.9	59.6±10.9	<2.2E-16^b^	61.6±11.6
e′ (cm/s)	9.41±2.13	8.63±1.83	<2.2E-16^b^	9.03±2.03
a′ (cm/s)	8.67±1.55	9.23±1.46	<2.2E-16	8.94±1.53
E/A	1.29±0.32	1.22±0.29	2.65E-7^b^	1.25±0.31
e′/a′	1.13±0.36	0.970±0.29	<2.2E-16^b^	1.05±0.34
E/e′	8.68±1.99	8.41±1.78	3.36E-4^b^	8.55±1.90
s′ (cm/s)	7.91±1.13	7.93±1.20	0.756	7.92±1.16
Vpe (m/s)	79.4±22.1	71.9±19.5	<2.2E-16^d^	75.7±21.2
DT (ms)	167±29	170±30	9.29E-4	168±30
Isovolumic Relaxation Time (ms)	81.8±13.8	90.2±12.9	<2.2E-16	85.9±14.0
LV Ejection Fraction (%)	64.8±6.7	62.5±6.4	<2.2E-16	63.7±6.7
LVEDD (mm)	44.9±3.9	49.4±4.4	<2.2E-16^b^	47.1±4.7
Sphericity (%)	56.9±6.4	57.2±6.5	0.246	57.0±6.4
Left Ventricular Mass (g)	124±31	179±40	<2.2E-16^b^	151±45
LVM index (g/m^^∧^^1.7)	54.2±13	69.0±15.0	<2.2E-16^b^	61.3±15.9
NTproBNP (pg/ml)	81.3±64.1	36.4±39.2	<2.2E-16^b^ ^d^	59.5±58.0

a : P-value for comparison between sexes using independent t-test (^b^:unequal variance) or chi-square test (^c^).

d : Data was log transformed before statistical testing.

LVM: allometrically scaled Left Ventricular Mass index, PBL TL: Periferal Blood Leukocyte Telomere Length, E & A: peak transmitral flow velocities during early (E) and late (A) diastolic filling, e′ & a′: peak movement speed of mitral annulus during early (e′) and late (a′) diastolic filling, DT: transmitral Deceleration Time, NTproBNP: N-terminal prohormone of brain natriuretic peptide, LVEDD: Left Ventricular End-Diastolic Diameter, s′: peak systolic mitral annulus movement speed, Vpe: pulse propagation velocity in early diastole.

### Diastolic function

**Figure 1:**
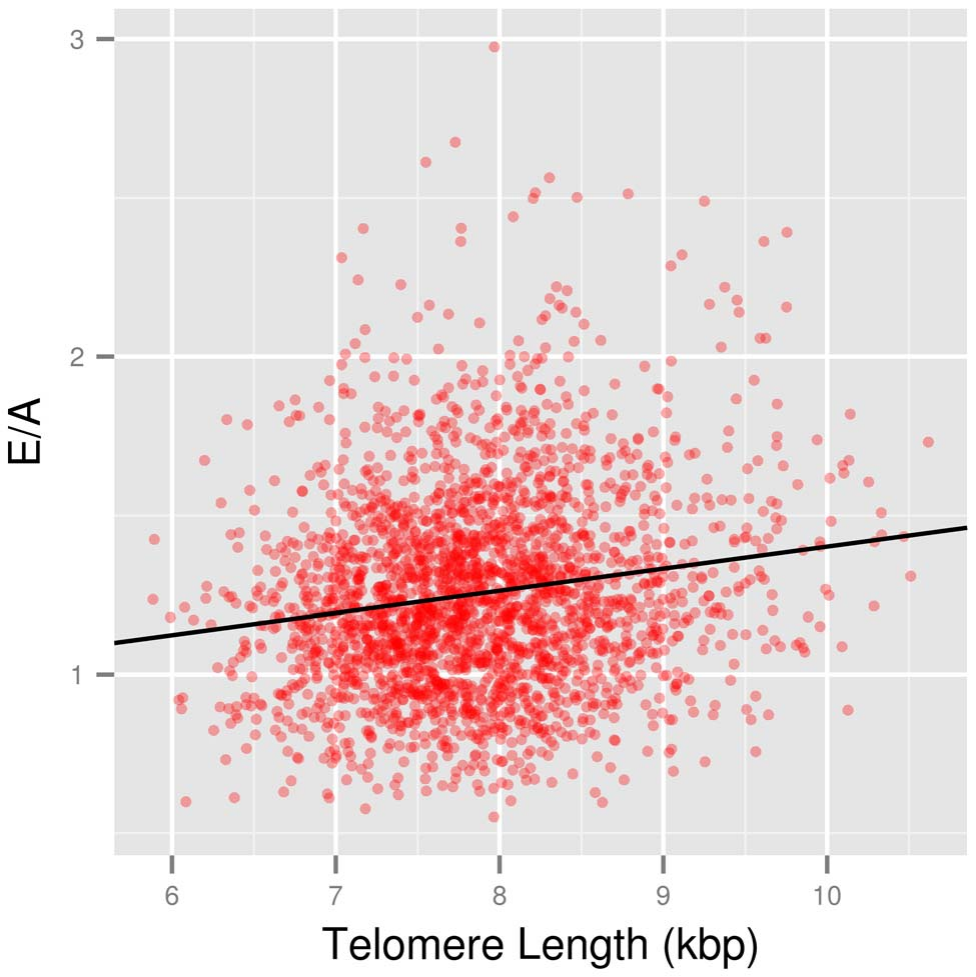
TL ∼ E/A. Scatterplot showing the unadjusted correlation between telomere length (TL) and the ratio of early (E) over late (A) mitral annulus movement speed (red) and the regression line (black).

Unadjusted models yielded positive linear associations between TL and E/A, e′/a′ (Fig. 1) and E/e′ (Table 2, Model 1). In successive (general linear) models, known major confounders of diastolic function were added, i.e. age and sex in Model 2, additionally heart rate (HR), systolic blood pressure (SBP) including use of antihypertensive drugs and body mass index (BMI) in Model 3. We did not remove non-significant independent variables from the proposed models in Table 2 for the individual response variables as removal of the non-significant terms did not alter the significance of the TL component. Addition of further potential confounders: LV sphericity, oxidative stress (oxidized-LDL cholesterol, serum uric acid) or inflammatory markers (high-sensitive CRP, IL-6), did not significantly alter the TL - diastolic dysfunction relationships when added to Model 3 as an independent variable (data not shown).

**Table 2:** The association of TL (kbp) with different parameters of cardiovascular function using general linear models.

Response variable (RV)		Model 1	Model 2	Model 3
E/A	b	0.0696	0.0249	0.0264
	P	2.83E-16	1.25E-03	8.09E-05
e′/a′	b	0.0764	0.0178	0.0182
	P	<2.2e-16	0.0281	0.0120
E (cm/s)	b	1.91	0.195	0.226
	P	1.09E-06	0.600	0.535
A (cm/s)	b	−1.60	−0.860	−0.905
	P	5.37E-07	4.62E-03	4.51E-04
e′ (cm/s)	b	0.397	0.050	0.043
	P	8.93E-13	0.304	0.341
a′ (cm/s)	b	−0.211	−0.068	−0.079
	P	5.77E-07	0.100	0.0403
E/e′	b	−0.158	−0.0214	−0.0124
	P	2.62E-3	0.677	0.794
DT (ms)	b	−1.86	−0.123	−0.302
	P	2.24E-02	0.881	0.712
IVRT (s)	b	−2.70	−0.941	−0.900
	P	2.32E-12	9.25E-03	0.0115
log(NTproBNP (pg/ml))	b	0.017	−0.004	−0.005
	P	0.119	0.657	0.617
LVM index (g/m^1.7^)	b	−2.37	−0.194	0.0228
	P	5.41E-08	0.616	0.943
LVEDD (mm)	b	−0.497	−0.164	−0.124
	P	1.11E-04	0.158	0.261
EF (%)	b	0.0216	−0,0183	0.0156
	P	0.906	0.922	0.933
s′ (cm/s)	b	0.018	−0.033	−0.038
	P	0.579	0.315	0.238

Model 1: RV ∼ TL.

Model 2: RV ∼ TL+Age+Sex.

Model 3: Model 2+Systolic BP+Heart Rate+BMI+Used Antihypertensive Drugs.

E & A: peak transmitral flow velocities during early (E) and late (A) diastolic filling, e′ & a′: peak movement speed of mitral annulus during early (e′) and late (a′) diastolic filling, DT: transmitral Deceleration Time, IVRT: Isovolumic Relaxation Time, NTproBNP: N-terminal prohormone of brain natriuretic peptide, LVM: allometrically scaled Left Ventricular Mass index, LVEDD: Left Ventricular End-Diastolic Diameter, EF: Ejected Fraction of end-diastolic volume, s′: peak systolic mitral annulus movement speed, b: effect size (unstandardized), P: P-value of TL component.

In all models the positive association between E/A and TL remained significant (see Table 2, P≤0.002) with Model 3 accounting for approximately 43.2% of E/A variability (2.64% of the total variability could be attributed to TL). Examining the data separately by sex, we found that, upon adjustment for confounders (Model 3), the association between E/A and TL was significant in both women (b = 0.030, P = 0.001) and men (b = 0.023, P = 0.014). A similar approach was adopted for the e′/a′ ratio. Results of the linear models were included in Table 2 and demonstrated significance of the adjusted associations for e′/a′ (Model 3: b = 0.018, P = 0.012). Looking at both sexes separately, TL was a significant independent variable in women (b = 0.022, P = 0.032), but only borderline in men (b = 0.016, P = 0.098).

We further examined the components of these ratios (E/A and e′/a′) separately to determine whether the associations were attributable to one of both components or whether the ratios contained additional information beyond the terms they consist of. Surprisingly, the results indicated that neither E (b = 0.226, P = 0.535) nor e′ (b = 0.043, P = 0.341) were correlated with TL (Model 3). There was however a correlation of TL with A (b = −0.905, P<0.001) and a′ (b = −0.079, P = 0.040).

**Figure 2:**
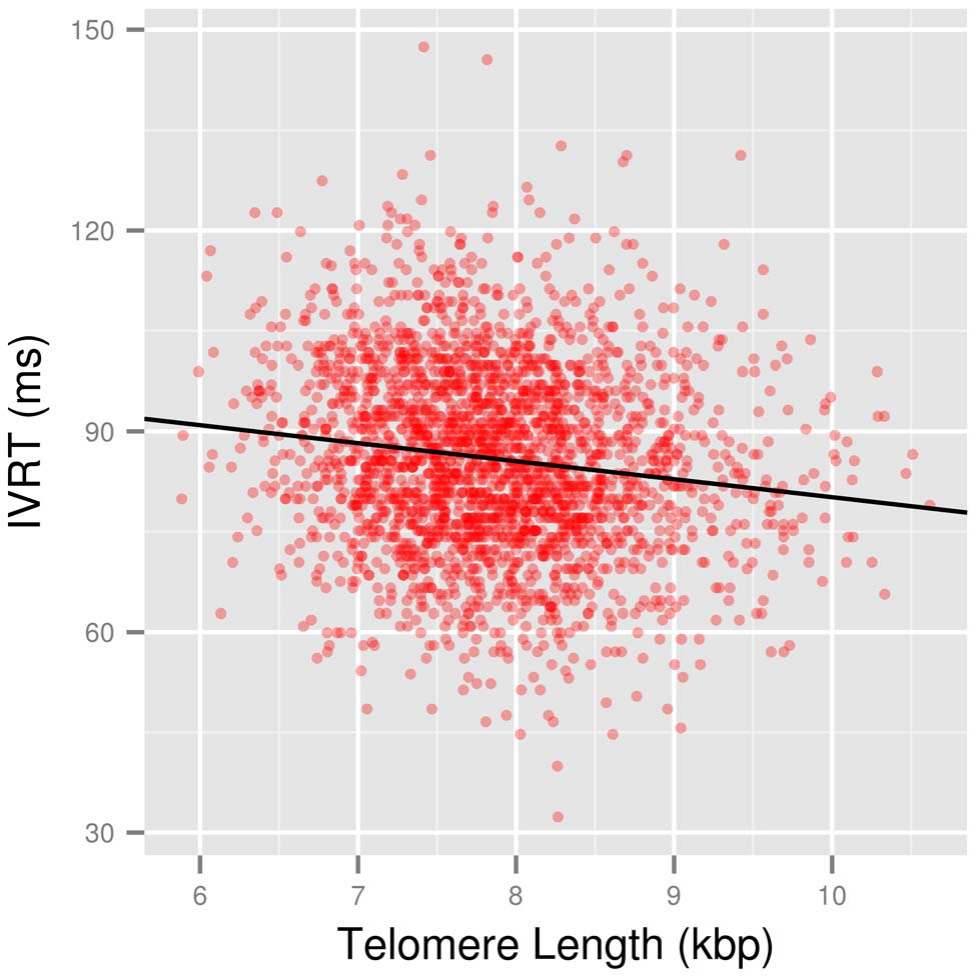
TL ∼ IVRT. Scatterplot showing the unadjusted correlation between telomere length (TL) and isovolumic relaxation time (IVRT) (red) and the regression line (black).

Accordingly, E/e′ was not correlated upon adequate adjustment (Model 3: b = 0.014, P = 0.775), neither were other indices of diastolic function: flow propagation velocity of the E-wave (Vpe; b = 0.212, P = 0.718), mitral inflow deceleration time (b = −0.302, P = 0.712) or duration of the atrial contraction (b = −0.028, P = 0.938). Only IVRT was inversely correlated with PBL TL (Fig. 2, Model 3: b = −0.900, P = 0.011). IVRT was also inversely correlated with PBL TL after age, sex and paternal-age adjustment (PBL TL as dependent variable; b = −2.92E-3, P = 0.010). Additionally, we found no significant association with brain natriuretic peptide (log(NT-proBNP): b = −0.005, P = 0.617) upon adjustment.

### Systolic function and LV structure

We could not document significant associations between TL and systolic function after adequate adjustment (Model 3, EF: b = 0.016, P = 0.933 and s′: b = −0.038, P = 0.238). Similarly no associations were found with LV structure assessed by LV end-diastolic diameter (LVEDD: b = −0.124, P = 0.261) and LV mass (b = −0.214, P = 0.801), body surface area adjusted LV mass (b = 0.010, P = 0.981) or allometrically height-adjusted LV mass (b = 0.023, P = 0.943).

### Arterial stiffness

No significant partial correlation was found between PWV and age-adjusted TL (cf. supra), a result that remained unchanged after additional adjustments (Model 3, b = −0.001, P = 0.971). The same is true for pulse pressure (PP) in the Asklepios population (Model 3, b = 255E-6, P = 0.999).

## Discussion

Our main finding is that we can extend a number of previously described associations between PBL TL and cardiovascular structure and function –usually detected in smaller, diseased cohorts - towards a middle-aged, apparently healthy population. After adjustment for confounders, we could not document an association with systolic function, cardiac structure or vascular stiffness. We do however document an intriguing association with certain parameters of LV filling.

### LV filling and diastolic function

As mentioned in the introduction, previous reports on the association between TL and indicators of diastolic dysfunction have led to apparently conflicting results. Quartiles of telomere length were shown to correlate with diastolic dysfunction in CAD patients (evaluated by E/A and pulmonary vein flow) [18]. In contrast both E/A-ratio and diastolic dysfunction were not correlated with PBL TL in a study of elderly subjects (>85 years) [19]. This can potentially be explained by the very different nature of the populations under study. Our data showed a significant association between PBL TL and both the E/A-ratio and the e′/a′-ratio and with IVRT [37], but not with the E/e′-ratio (see Table 2 for details). E/e′ is mainly used as an indicator of elevated filling pressures, reflecting more advanced diastolic dysfunction not likely to be present in healthy subjects [38]. Concurrently, NT-proBNP, a biochemical marker reflecting elevated filling pressure and diastolic dysfunction [39], was not associated with PBL TL after correction for confounders (Model 3).

Analysis of the terms constituting the E/A and e′/a′ ratios in this population suggests that TL was associated with atrial contraction (A and a′), rather than with parameters that could reflect myocardial relaxation or filling pressure, such as E and e′, or reflect myocardial stiffness, such as shortened mitral deceleration time [40]. As previous publications only described correlations with E/A but not with the individual components, we cannot tell whether this was the case in other study populations.

Relaxation and stiffness induce opposite effects on mitral E and A and hence on E/A [40]. The present data do not provide sufficient evidence for an independent association between PBL TL and LV relaxation or LV stiffness in the general middle-aged population. Although there is an association with longer IVRT after correction for heart rate and blood pressure, it is difficult to attribute this to myocardial relaxation without a persisting association with the best validated determinants e′ and Vpe [41]. The persisting associations with atrial contraction flow velocity (A) and with the simultaneously occurring annular velocity (a′) then most likely affects compound measures such as E/A and e′/a′. One could speculate that increased IVRT and enhanced atrial contraction represent an early and subtle delay of myocardial relaxation, which is not yet apparent in other measurements. We therefore would describe the findings as correlation between PBL TL and altered filling pattern without sufficient evidence for diastolic dysfunction. It is noteworthy though that the associations with PBL TL were stronger in women than in men, an interesting finding in light of the increased occurrence of diastolic HF in women [42].

### LV systolic function, structure and vascular stiffness

In this population without overt cardiac disease, we report that PBL TL was not associated with minor changes in systolic parameters such as EF and tissue Doppler movement speed of the septal mitral annulus (s′). Increased LV mass correlates with increased all-cause and cardiovascular mortality [43] and a positive correlation has been described between LVM and PBL TL [20]–[22]. However, our findings do not support an association between PBL TL and LVM, body surface area scaled LVM or height-scaled LVM in the context of a relatively young population. Two other studies also failed to detect an association between TL and LVM index or LV hypertrophy in two older populations (∼65 years) [18], [26]. These findings and the fact that the former studies do not agree on whether normotensive or hypertensive patients show a TL – LVM correlation, lead us to the conclusion that there is likely an as of yet unidentified confounder at work. A third study found no cross-sectional or longitudinal associations between PBL LTL and cardiac measurements including LVM (adjusted for body surface area) [27].

With respect to vascular stiffness, no significant associations between PWV (or PP) and TL were found in either sex. Previous studies have reported this association to be significant in men [15], [44]. The discordance may be attributable to age and health characteristics of the respective populations. Indeed these populations were featured by a higher mean age (∼10 years) and a higher mean PWV (60% higher) compared to the Asklepios study albeit with a different measurement protocol for PWV.

### Limitations

The Asklepios data set does not yet include any longitudinal information thus limiting it to all the drawbacks associated with cross-sectional study designs. Particularly claims of causality can not be made with cross-sectional data alone. Despite the extensive characterization of the Asklepios study, there are some descriptors of CV function which were not measured (e.g. pulmonary venous flow). We cannot exclude the involvement of these factors.

### Mechanistic insights

There are two general models in which TL is tied to cardiovascular health. The first states that telomere shortening is a primary driver of (cardiovascular) ageing. In support of this model our findings indicate that the associations between some parameters of LV filling and telomere length, are not limited to (chronic) HF patients, but may already be present in a young to middle-aged, apparently healthy population and are more pronounced in women. It is tempting to speculate that telomere biology could be mechanistically involved in the early pathogenesis of diastolic dysfunction and possibly HF by extension. As a matter of fact, in mice, knock-out of the telomere elongating enzyme telomerase, resulted in shortened telomere length over several generations which was associated with the development of overt chronic HF [45]. However, telomere biology in mice cannot be easily transposed to humans and additional experiments would be absolutely necessary to pinpoint the exact mechanisms.

In our data, TL was clearly correlated with E/A, e′/a′ and IVRT but not with other indices of diastolic function. We further explored the relationship between PBL TL and other cardiac and hemodynamic parameters such as sphericity of the ventricle, Vpe, duration of the A-wave and deceleration time (data not shown). No significant associations were found that could help provide clues as to the mechanism by which PBL TL and diastolic function might be linked.

The second model states that TL is merely an epiphenomenon, an indicator influenced by conditions in the ageing body. Accelerated telomere attrition in subjects with mildly impaired diastolic function could also be caused by oxidative stress and inflammation, two factors that are known to affect diastolic function as well as telomere length [14], [23], [46]–[48]. In this case it might be expected that the addition of oxidative stress and inflammation would cause reduced significance for TL. However, additional markers (CRP, oxidized LDL, IL-6 and serum uric acid) did not substantially alter the significance of the PBL TL component relative to Model 3 in Table 2 (data not shown). It should be noted though that these markers only reflect point measurements of oxidative stress and inflammation, which are variable by nature, whereas telomere length has been hypothesized to reflect their cumulated effects (reviewed in De Meyer et al. [4]).

Our data, at present, is insufficient to determine the more likely model. Some of the non-replicated associations might still become apparent with ageing, assuming that a certain threshold of telomere attrition needs to be reached before it has a measurable effect on cardiovascular stiffness or visa versa.

## Conclusions

Our results show that several parameters of cardiovascular structure and function which have been associated with TL, fail to replicate in a well-phenotyped middle-aged population sample. However, PBL TL is associated with subtle changes in certain parameters of LV filling in this population and these associations are more apparent in women. Further investigation of the underlying biological mechanisms is warranted to provide insights into the relationship between PBL TL, diastolic function and cardiovascular health.
